# The relationship between social odour awareness and emotional contagion susceptibility in females

**DOI:** 10.1177/03010066241270209

**Published:** 2024-08-12

**Authors:** Alexander W.J. Freemantle, Lorenzo Dante Stafford

**Affiliations:** 200435 University of Portsmouth, UK

**Keywords:** social odours, odour awareness, emotional contagion, smell

## Abstract

Previous research has shown a strong link between our sense of smell and emotion. More recently, the importance we attach to olfaction has been found to relate to our susceptibility to ‘catch’ the emotions of others. We explore this further by examining the relation between a newly developed measure of olfaction (social odour scale, SOS), which measures awareness of social odours, and emotional contagion susceptibility in female participants. The study therefore aimed to test the strength of this relationship and also help validate the English language version of the SOS. Female (n = 148) participants completed an online study that measured odour awareness [SOS; important of odour questionnaire, IOQ] and emotional contagion (EC). We found that the English version of the SOS yielded high reliability and supported the previous factor structure of the scale; additionally, we demonstrated a strong association between the SOS and IOQ which provides criterion validity for its usage. The study also revealed that whilst both the SOS and IOQ were positively associated with EC, the SOS was the more accurate predictor. These findings provide further validation for the use of the SOS and suggest that our subjective awareness of olfaction, especially concerning ‘social odours’ is an accurate predictor of emotional contagion.

Emotional contagion is the mechanism through which emotions can be interpersonally transferred in social situations (Hatfield et al., 1994). Often called ‘primitive’ emotional contagion, the unconscious interpersonal transfer of emotion can be facilitated by the detection and response to an individual's tone of voice, facial expressions, body language and other microbehaviours (Barsade et al., 2018). This interpersonal emotional transfer has been shown to have profound implications for social interactions through influencing the emotional states, attitudes, and behaviours of individuals within a social group (Barsade, 2002). For instance, in the context of competitive sports, it has been shown that the emotions of a team competing together can aggregate through prolonged social interaction (Freemantle et al., 2022a). More broadly, interpersonal emotional influence has also been identified in work environments. For example, evidence has been presented that shows paramedics’ own emotional states affecting the emotions and behaviours of their work partners as they complete a shift together (Freemantle et al., 2022b). The effects of emotional contagion have therefore been identified in a range of social situations, however, researchers have suggested that individual differences and other contextual factors, such as power and the social characteristics of the relationship, influence individuals’ experiences of these interpersonal emotional reactions (Hatfield et al., 2014). Identifying individual differences in emotional contagion susceptibility (ECS) is important as the ability to accurately perceive and respond to emotional cues from other individuals can be crucial in demonstrating effective social functioning, empathy and emotional regulation (Decety & Moriguchi, 2007). As such, ECS scales such as the emotional contagion scale (Doherty, 1997) can be used both as an indicator of emotional intelligence and as a measure of the impact of emotional stimuli.

Emotional contagion can be facilitated through means of visual and auditory modalities (Herrando & Constantinides, 2021); however, the olfactory system has also been shown to assist in this emotional transfer through the unconscious olfactory recognition of human chemosignals (Chen & Haviland-Jones, 2000). Human chemosignals, the chemicals in bodily secretions, can be found in tears (Gelstein et al., 2011), saliva and sweat (de Groot et al., 2012). Chen and Haviland-Jones (2000) showed that participants were able to distinguish between a happy body odour, a fearful body odour and a control odour, thus providing evidence that body odours contain an identifiable chemosignal profile that differs across different experienced emotions. This was a first step in demonstrating that emotional information could be conveyed within social odours. The unconscious detection of these emotional chemosignals has been shown to also enable the interpersonal transfer of both positive and negative emotions through the process of emotional contagion. De Groot et al. (2015) showed that the olfactory detection of the chemosignal profile of happy body odours caused receivers to ‘catch’ this positive emotion and subsequently exhibit happier facial expressions. Further evidence, using a range of methodologies (see de Groot et al. 2017 for review), has been presented for the interpersonal transfer of additional emotions, such as fear (de Groot et al., 2012), sadness (Gelstein et al., 2011) and aggression (Mutic et al., 2016) through olfactory facilitated emotionally contagious and reciprocal reactions. As such, the research suggests that certain odours possess social value and can therefore convey important information about an individual's identity, emotional states, and social cues (Lundström & Olsson, 2010). These odours can also subsequently lead to emotional contagion and the ‘catching’ of another's emotional state.

In the literature, little attention has been given to the role of individual differences when studying emotional contagion in the domain of olfaction. One study that did examine this phenomenon, measured individuals’ sense of smell using objective tests (threshold and identification) and subjectively (important of odour questionnaire [IOQ]) and then had individuals complete a task together (Freemantle et al., 2022c). The key finding was that subjective rather than objective measures of odour function related to emotional aggregation. Specifically, the results showed a positive association between the frequency with which participants reported using their olfactory sense, measured using the importance of olfaction questionnaire (IOQ; Croy et al., 2010), and their ability to catch emotions from others when completing a collaborative task together. However, the Freemantle et al. (2022c) study used only the IOQ as a measure of olfactory utility and did not include a more specific subjective measure of social odour. Additionally, Freemantle et al. (2022c) did not measure emotional contagion and instead analysed aggregated emotion measures. Understanding how an individual's reaction to specifically social odours affects interpersonal emotional processes would help to provide stronger evidence of the role of olfactory systems in emotional contagion and begin to reveal how this process may be managed and trained. Social odour awareness encompasses an individual's ability to detect, recognize, and discriminate between different social odours, providing valuable insights into the mechanisms underlying olfactory processing in a social context (Lundström & Olsson, 2010). Examining individual differences in social odour awareness is relevant for understanding the influence of olfactory cues on social interactions and emotional contagion. Work conducted by Lundstrom et al. (2006) demonstrated individual differences in the detection and processing of social odours and the difference with which social odours were processed when compared to common odours. Similarly, the literature suggests that there exist individual differences in social odour processing and therefore some individuals may process social odours quicker, more accurately, or rely upon the process more in their day-to-day lives (Mantel et al., 2019; Sorokowska et al., 2018). However, these studies have not investigated the effect that these differences may have upon interpersonal emotional processes.

Individual differences in social odour response may also develop as a result of the different neurological mechanisms with which individuals respond to social odours (Lundstrom & Olsson, 2010). Evidence suggests that some individuals exhibit a heightened social odour processing, allowing them to effectively perceive and interpret social odour cues, while others display lower sensitivity or limited awareness of such olfactory stimuli (Ferdenzi et al., 2016).

The development of the social odour scale (SOS; Dal Bo et al., 2021) provides a reliable measure for assessing an individual's level of social odour awareness. The SOS includes items that assess an individual's awareness of different social odours and their ability to discriminate between them, and therefore is more specific in its focus on social odours than questionnaires such as the IOQ which includes attitudes toward common odours. In addition, the authors of the Social Odour Scale suggest that the utility of the SOS extends to clinical populations and the scale's use within research may be able to characterise and increase our understanding of complex psychological disorders (Dal Bol et al., 2021). However, to date, the English language version of the SOS has not been validated and therefore an additional aim of the current research is to understand the reliability and factor structure of the English version of the scale.

Emotional contagion is influenced by various factors, including personality traits, empathy, and emotional regulation (Hatfield et al., 1993; Hatfield et al., 1994). However, the potential relationship between social odour awareness and susceptibility to emotional contagion remains relatively unexplored (see Freemantle et al., 2022c). Investigating the connections between these processes can provide valuable insights into the influence of olfactory cues on emotional contagion in social situations. It is possible that individuals with higher social odour awareness may be more attuned to olfactory cues in their social environment, thereby exhibiting higher susceptibility to emotional contagion and being affected more by the emotions of others. By investigating the relationship between social odour awareness and susceptibility to emotional contagion, we can shed light on the unique contribution of olfactory communication to emotional contagion processes.

The present study therefore aimed to examine the relationship between emotional contagion and the olfactory system from both a general (IOQ) and social (SOS) perspective. Due to the observed differences in EC between males and females (Doherty et al., 1995), we decided to restrict the study to females, who completed the EC, IOQ and SOS. The increased tendency to experience emotional contagion in females (Doherty et al., 1995), as well as the improved odour function (Hummel et al., 2007), would suggest that female participants may be more likely to demonstrate a detectable relationship between social odour awareness and ECS. As such, this study recruited female participants in the first instance. If a relationship is identified, then a future study could extend to both male and female participants. We hypothesise that both the IOQ and SOS will positively predict an individual's ECS and we tentatively predict that SOS will be a more accurate predictor.

## Materials and Methods

### Participants

One hundred and forty-eight females took part in the study; ages ranged from 18 to 45 (*M* = 31.9; *SD* = 6.6). All participants were recruited via prolific and reported being nonsmokers and nonvapers and none suffered from anosmia. The required sample size was calculated using GPower for a multiple regression statistical test with an alpha level of 0.05, a power of 0.95 and 3 total predictor variables. This sample size was adequate to identify a small to medium effect, which is similar to other previously conducted studies. The study gained ethical approval through the University's Faculty of Humanities and Social Sciences Ethics Committee (FHSS 2022-053), which complies with the Declaration of Helsinki for Medical Research involving Human Subjects; all participants provided written informed consent. The data were collected in April 2023. Since recruitment was via prolific where individuals are given a unique code, neither researcher had access to information that could identify individual participants during or after data collection.

### Design

The study used a regression design exploring the following main variables: Social Odour Scale scores, Importance of Olfaction Scores and Emotional Contagion Scale scores.

### Measures

#### Social Odour Scale

The Social Odour Scale (SOS: Dal Bo et al., 2021) was used to assess the degree to which participants attend to social cues in odours. An English version of the scale was used, which was translated from the original Italian version. This is a 12-item questionnaire which consists of three subscales. The three subscales relate to an individual's awareness of the social odours of their partner, family members and strangers. The SOS uses a five-point Likert scale ranging from 1 (*I totally disagree*) to 5 (*I totally agree*). A mean score for each subscale and a total SOS mean score was calculated.

#### Individual Significance of Olfaction Scale

The IOQ (Croy et al., 2010) assessed the participants’ attitudes towards the utility and importance of their own olfactory system. The IOQ consists of the three subscales application, association and consequence. The application subscale measured an individual's subjective belief as to the extent to which they use their sense of smell in their daily lives. The association subscale measured how important odour is in evoking memories, emotions and values. Finally, the consequence subscale represented the extent to which individuals use olfaction to make daily decisions. The IOQ is assessed using a four-point Likert scale ranging from 1 (*I totally disagree*) to 4 (*I totally agree*). The means of each subscale were then averaged to create a mean IOQ score.

#### Emotional Contagion Scale

The participants’ susceptibility to experiencing emotional contagion was measured using the emotional contagion scale (Doherty, 1997). The emotional contagion scale is a 15-item scale that is categorised into five subscales. The five subscales represent the participant's susceptibility to experience emotional contagion for five different emotions: love, anger, happiness, fear and sadness. A four-point Likert scale is used within the emotional contagion scale which ranges from *Always true for me* to *Never true for me.*

### Procedure

The survey was hosted on Qualtrics, an online survey platform, and the study recruitment and completion were conducted via prolific. Prolific users were only recruited to the study if they reported being nonsmokers and nonvapers, did not suffer from anosmia, were female and were aged between 18 and 45. The participants were provided with the information sheet and offered the opportunity to ask the researcher questions about the study before deciding if they wished to take part and completing the informed consent form. Demographic information was then collected from the participants before they were presented with the three questionnaires. The questionnaires included the SOS (Dal Bo et al., 2021), the emotional contagion scale (Doherty, 1997) and the IOQ (Croy et al., 2010). The order of these questionnaires was randomised so as to limit order effects. There was also an attention test included during the procedure to check for participant attention. In this attention test, participants were asked to select a specific correct answer from a list of options. If any participants answered this attention check incorrectly then they were removed from the study. All of the 148 participants answered this attention test correctly. Once these questionnaires were completed, the participants were provided with the Debrief form and their participation was over. Each participant received a pro-rata payment of £9.00 per hour for their time through the prolific system.

### Statistical Analysis

There were 148 participants who took part in this study. Following the removal of outliers and participants who had completed the questionnaires in an unusually quick time (< 180 s), data from 139 participants were included in the analysis. The assessment of the relationship between social odour awareness and ECS was achieved using a multiple regression analysis technique in SPSS Version 28. The entry method regression models assessed whether the individuals’ scores on the emotional contagion scale could be predicted by their SOS scores and their IOSs, while controlling for the participants’ age.

The criterion validity and factor structure of the English version of the SOS (Dal Bo et al., 2021) were assessed using a correlation analysis involving the SOS and the IOQ (Croy et al., 2010). This analysis formed an assessment of the SOS criterion validity because the SOS and IOQ scales measure similar concepts. A principal component analysis (PCA) was also completed to assess the SOS's factor structure and general structural validity.

## Results

### Descriptive Statistics

#### Social Odour Awareness

The SOS was used to assess participants’ social odour awareness and response. The mean sum score for participants was 32.70 (*SD* = 7.53). The partner subscale presented a mean score of 11.81 (*SD* = 3.98), the familial subscale presented a mean score of 9.12 (*SD* = 3.31) and the stranger subscale presented a mean score of 11.80 (*SD* = 3.20). The SOS presented a Cronbach's alpha score of .808.

#### Individual Significance of Olfaction

The mean IOQ score for the participant group was 2.13 (*SD* = .33) and the Cronbach's alpha was .885.

#### Emotional Contagion Scale

The mean emotional contagion scale score was 1.94 (*SD* = 0.43) and the Cronbach's alpha was .851. The mean scores for the subscales of the emotional contagion scale were shown to be similar across the participants. The mean score for the love subscale was 1.87 (*SD* = 0.66), the mean score for happiness was 1.74 (*SD* = 0.47), the mean score for fear was 1.88 (*SD* = 0.58), the mean score for anger was 2.27 (*SD* = 0.58) and the mean score for sadness was 1.96 (*SD* = 0.60).

#### Factor Structure of the SOS

The factor structure of the English version of the SOS had not previously been assessed, therefore a PCA assessment was run using SPSS Version 28. Assumptions testing was completed and the data showed a Kaiser-Meyer-Olkin value of 0.783 and Bartlett's test of sphericity was significant. This ensured that PCA could be conducted. The PCA was conducted using a varimax rotation and the extraction of factors was based upon eigenvalues > 1 and by using a scree plot. The rotated component matrix showed that three factors were extracted. The total percentage of variance explained by these three factors was 64.43%. These three factors largely fit the current three subscales of the Social Odour Scale. The only exception was that Item 5 (*I can be attracted to someone for their body odour*) showed a larger factor loading for the Family subscale than the Partner subscale. Nevertheless, the factor loadings for this item in relation to both subscales were above 0.4, therefore indicating that the item may be relevant for both subscales. The presented factor loadings can be seen in Table 1. Overall, these results support the current structure of the SOS.

**Table 1.** Factor loadings of the social odour scale.

Emotion	Factor 1 (family)	Factor 2 (partner)	Factor 3 (stranger)
Item 3	0.778		
Item 4	0.776		
Item 1	0.747		
Item 2	0.735		
Item 5	0.573	0.411	
Item 8		0.893	
Item 7		0.879	
Item 6		0.755	
Item 10			0.844
Item 11			0.806
Item 12			0.800
Item 9			0.547

*Note.* Extraction method: principal component analysis. Rotation method: varimax with Kaiser normalization.

#### Criterion Validity

The SOS (Dal Bo et al., 2021) and the IOQ (Croy et al., 2010) both assess individual's attitudes towards their olfactory system, albeit the SOS focuses solely on the role of olfaction in social situations while the IOQ includes items related to both social and nonsocial odours. As such, the relationship between the SOS scores and the IOQ scores can be used, to an extent, as an indicator of the SOS's criterion validity in assessing social odour awareness. The strength of the relationship between these two variables, assessed using a correlational analysis, was found to be .569 (*p *< .001). This indicates a large correlation between the scores of the two scales (Cohen, 1988) and good criterion validity of the SOS.

#### Regression Analysis

**Figure 1.**
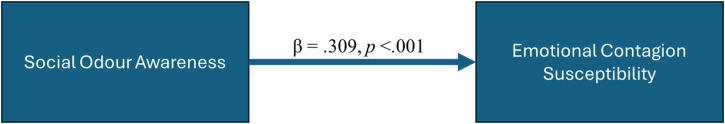
Regression model for social odour awareness predicting emotional contagion susceptibility.

A number of regression models were run to assess the relationship between attitudes towards social olfaction and ECS. First, a model investigating the relationship between the social odour scale scores and the emotional contagion scale scores was run. The model was found to be significant, *R* = .309, *F*(1, 137) = 14.50, *p *< .001, and the individuals’ social odour awareness scores positively predicted their ECS (β = .309, *p *< .001) (Figure 1). A significant effect of social odour awareness on ECS was also presented when controlling for the participants’ age, *R* = .330, *F*(2, 136) = 8.309, *p *< .001. The participants’ age did not have a significant effect when included in that model (β = −.115, *p *= .159).

**Figure 2.**
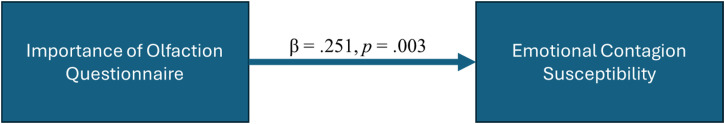
Regression model for importance of olfaction questionnaire scores predicting emotional contagion susceptibility.

A similar regression model was run which assessed the relationship between the participants’ IOQ score and their susceptibility to experience emotional contagion. The model showed that the IOQ scores significantly predicted the ECS scores, *R* = .251, *F*(1, 137) = 9.217, *p *= .003, β = .251, (Figure 2).

**Figure 3.**
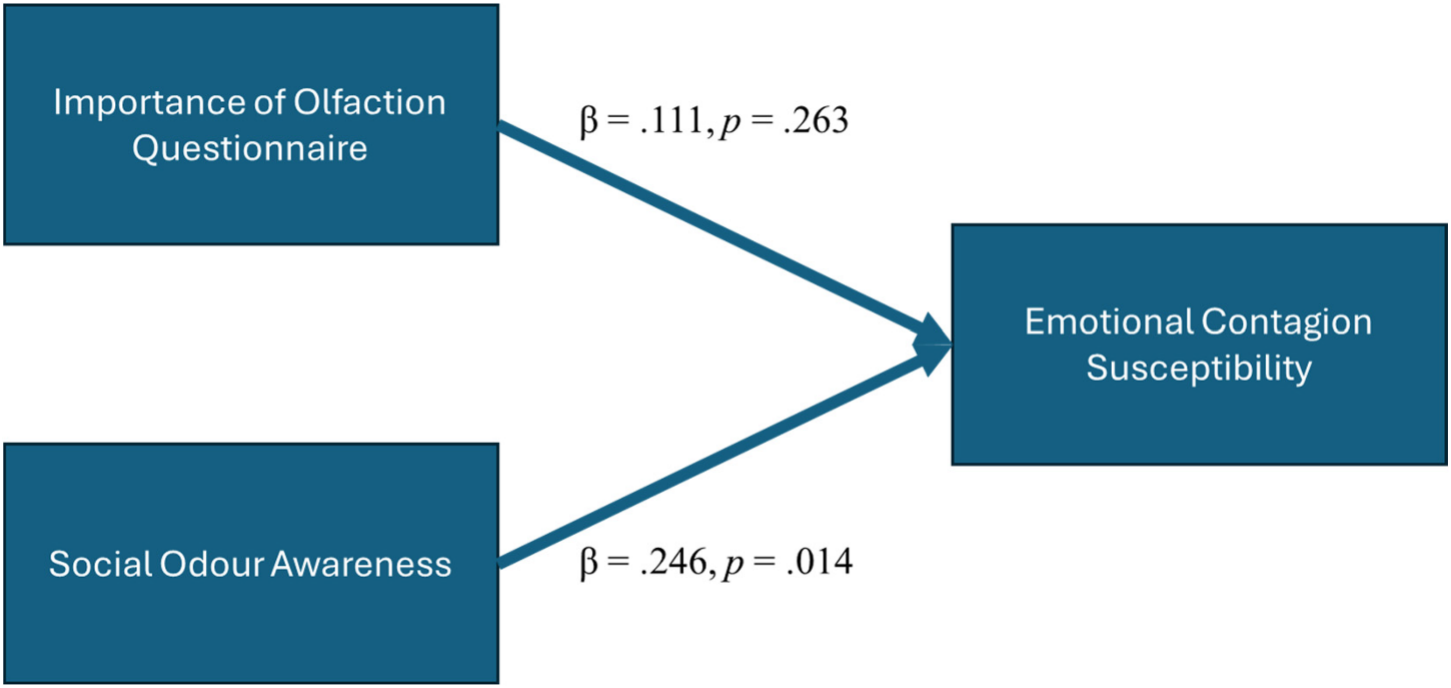
Regression model for social odour awareness and importance of olfaction questionnaire predicting emotional contagion susceptibility.

A multiple regression model which included both the importance of the olfaction questionnaire and the SOS showed that the SOS scores significantly predicted the ECS, β = .246, *F*(2, 136) = 7.898, *p *= .014, while the IOQ score was not significant in this model (β = .111, *p *= .263) (Figure 3). This suggests that SOS is the more accurate predictor of EC.

## Discussion

The present study investigated the relationship between individuals’ attitudes towards the use of their sense of smell in social and non-social situations and the tendency with which they experience emotional contagion. There were two main analyses completed during this study. First was an assessment of the factor structure and criterion validity of the English-translated SOS (Dal Bo et al., 2021). This was the first use of the translated scale and a PCA showed that the factor structure of the English SOS was adequate and effectively demonstrated the structure of the three key factors of the original SOS (family, partner and stranger). In addition, an assessment of the correlation between participants’ SOS scores and the IOQ scores (Croy et al., 2010) showed a strong positive correlation. Owing to the similarity in concepts between the IOQ and the SOS, albeit the IOQ is predominately focused on individuals’ attitudes towards common odours with some consideration of social odours, these results indicate that the SOS demonstrates good criterion validity. In sum, the results from this study offer further support to the validity of the SOS (see Dal Bo et al., 2021) through an additional assessment of the factor structure and the first comparison of the SOS with the IOQ. Additionally, the validity of specifically the English-translated SOS has been found to be satisfactory in this instance. Further use of the SOS in future studies investigating individuals’ attitudes to social odour is encouraged.

Secondly, we used regression analyses to assess the extent to which scores on the SOS and the IOQ impacted an individual's susceptibility to experiencing emotional contagion (measured using the emotional contagion scale, Doherty, 1997). We found that the participants’ SOS scores were able to significantly predict their tendency to experience emotional contagion. Scores on the IOQ also significantly predicted emotional contagion scale scores. Nevertheless, participants’ SOS scores presented a higher standardised beta score than that of the IOQ scores when predicting ECS. Additionally, when both variables were included as predictors in the same model, the SOS scores significantly predicted the emotional contagion scale scores while the IOQ scores did not.

These results here help to extend our understanding in a number of important ways. First, the significant positive relationship between the olfactory variables (the SOS and the IOQ) and the measure of ECS (the ECS) provides further evidence that the olfactory system is involved in the interpersonal transfer of emotion. These findings support previous evidence from physical odour presentation studies, such as those with body odour collection and presentation (de Groot et al., 2017), and experiments utilising emotional self-assessments and objective olfactory measures (Freemantle et al., 2022c). The present study used an alternative, more practical methodological approach in the form of self-assessed attitude questionnaires, yet a relationship between olfaction and interpersonal emotional processes was still found. The second key finding from this study was that the SOS variable demonstrated a stronger effect on the ECS scores than the IOQ variable and, when entered into the same regression model, only the SOS was a significant predictor of ECS. We posit that these results were found as a result of the focus of the SOS on individuals’ responses to specifically social odours, while the IOQ largely measures attitudes to nonsocial, common odours such as fruit at a supermarket and shampoo. In contrast, the SOS was directly developed to assess an individual's attitude towards the odours that they experience in social situations (e.g., *I get quickly annoyed by the odour of strangers*). Therefore, it was expected that the SOS scores would be most relevant to any olfactory-facilitated interpersonal emotional transfer. Indeed, the evidence that the SOS results demonstrated better predictive power for ECS supports theories that the olfactory system is involved in emotional contagion processes (de Groot et al., 2017). As olfactory chemosignals originate from individuals in social situations, it is perhaps not surprising that individuals who detect and pay more attention to these social body odours are more susceptible to their effects. Freemantle et al. (2022c) observed a relationship between the IOQ and interpersonal emotional processes when dyads worked on a collaborative task; the results here extend those findings by demonstrating that it is the SOS rather than the IOQ which is a better predictor of an individual's tendency to catch others’ emotions. As such, it is recommended that the SOS is used in future studies when assessing olfactory attitudes and processes that may be relevant in social situations.

The results presented in this study support previous evidence which demonstrated the strength of individual differences in social odour awareness (Sorokowska et al., 2018). The findings suggest that individual differences exist when investigating the impact that the presentation of social odours can have on an individual's own emotional state. SOS scores were found to be a significant predictor of ECS, therefore demonstrating support for the role of olfaction in emotional contagion processes and showing the value of using the SOS as a measure of individual differences in detecting and processing human chemosignals.

The current study also builds on work on the relationship between individual differences and emotional chemosignals. For instance, Haviland-Jones et al. (2016) found that individuals could be categorised (non-detector/detector/super detector) in their ability to detect the emotional (fear/happy) body odours of others. The results here extend that work by demonstrating the utility in individuals’ subjective awareness of odours. In particular, they reveal that from the opposite perspective, the capture (rather than detection) of emotions can be predicted by ‘social’ odour perception.

The relationship between individuals’ socioemotional skills and social olfactory capabilities was also demonstrated by Zhou and Chen (2009) who found that cognitive and visual-emotional processing predicted the identification of socially relevant body odours. However, while both studies identify potential individual differences in olfactory facilitated emotional responses, the present study demonstrates the effect of a social olfactory measure on specifically an ECS assessment rather than just social odour identification. As such the eventual effect of the social odour detection is also considered here.

In terms of limitations, we acknowledge that as we tested females only, we are unsure whether the same findings would apply to males. The rationale for restricting the study to females was based on previous work showing differences in EC between males and females (Doherty et al., 1995), and additionally, the known differences in odour function and emotional odour identification (Chen & Haviland-Jones, 2000; Hummel et al., 2007). Hence, if an effect was present, then it was expected that female participants, rather than male participants, would demonstrate a stronger association between their social odour awareness and their ECS. This assumption may have been correct as a significant effect was identified in the present study. Nevertheless, future research should be conducted to assess the relationship between social odour awareness and ECS with participant cohorts that include both male and female participants.

Moreover, despite recruiting female participants, we did not measure an additional relevant control variable such as menstrual cycle phases. Evidence has been presented for the effect that menstrual cycle phases can have on olfactory capability, perhaps most notably socially relevant odour sensitivity (Nabergoj et al., 2020; Novakova et al., 2014), as well as social-emotional responses (Derntl et al., 2013). Following evidence from the current study which suggests that a relationship between ECS and social odour perception exists in a female population, future studies involving solely female participants should look to utilise menstrual cycle phases as an additional predictor to ascertain what impact this factor may have upon the strength of the relationship.

The methodology adopted in the present study utilised a questionnaire design which examined the relationship between the attitude measures. This methodology allowed for a large number of participants and ensured that the validity and reliability of the SOS could be assessed. However, previous studies have instead used controlled investigations involving the collection and presentation of body odour when examining the role of olfaction in interpersonal emotional processes. For instance, instead of assessing real-world emotional aggregation as a measure of emotional contagion (e.g., Freemantle et al., 2022c), the present study used a questionnaire that assessed an individual's tendency to experience emotional contagion in social situations. Similarly, the present study used a questionnaire to assess the participants’ attitudes towards their exposure to social odours rather than assessing the participants’ physical detection and response to social odours (e.g., Haviland-Jones et al., 2016). Although the chosen methodology was relevant to the aims of the present study, future investigations are encouraged to explore this area using objective odour tests.

In summary, the present study provided evidence for social odour awareness scores being able to predict an individuals’ ECS. This extends upon the literature as previous findings have not been able to identify individual differences in olfactory-facilitated interpersonal emotional processes to such an extent. As such, this study further supports the notion that the olfactory system exhibits influence on interpersonal emotional processes such as emotional contagion and indicates that measures of attitudes towards certain social odours should be considered as a relevant factor in study designs and real-world social situations.
